# Dynamic changes in serum albumin, prealbumin, and retinol-binding protein after head and neck cancer surgery: a retrospective cohort study

**DOI:** 10.3389/fmed.2026.1859391

**Published:** 2026-07-03

**Authors:** Linjian Huang, Simin Deng, Xiahan Sheng, Hanwen Chu

**Affiliations:** Department of Oral and Maxillofacial Surgery, The Second Affiliated Hospital, Zhejiang University School of Medicine, Hangzhou, Zhejiang, China

**Keywords:** head and neck malignant tumors, perioperative nutrition, nutritional intervention, prealbumin, retinol-binding protein

## Abstract

To investigate the dynamic changes in serum albumin (ALB), prealbumin (PA), and retinol-binding protein (RBP) before and 1 week after surgery in patients with head and neck malignancies, and to evaluate the effects of intensive nutritional intervention on postoperative nutritional recovery, inflammatory response, and nutritional target achievement. A total of 200 patients with head and neck malignancies who underwent surgery between January 2023 and December 2024 were retrospectively analyzed. Patients were divided into a control group (*n =* 100) receiving conventional nutritional support and an intervention group (*n =* 100) receiving intensive nutritional intervention. Serum ALB, PA, and RBP levels were measured preoperatively and on postoperative day 7. Inflammatory markers (CRP and NLR), postoperative recovery indicators, and energy and protein target achievement rates on postoperative days 3 and 7 were also compared between the two groups. There were no significant differences in baseline indicators between the two groups. One week after surgery, the intervention group showed higher levels of ALB, PA, and RBP compared with the control group (*p* < 0.05). CRP and NLR levels were significantly lower in the intervention group (*p* < 0.05). The intervention group also had a shorter time to first oral intake and shorter hospital stay (*p* < 0.05), while the incidence of complications showed a decreasing trend. In addition, the rates of energy and protein target achievement on postoperative days 3 and 7 were higher in the intervention group than in the control group (*p* < 0.05). Intensive nutritional intervention was associated with improved perioperative nutritional status, attenuated inflammatory response, and better early postoperative recovery in patients with head and neck malignancies. Its benefits were reflected not only in improved nutritional biomarkers but also in higher rates of early postoperative nutritional target achievement. PA and RBP appeared to be more sensitive indicators than ALB for dynamic perioperative nutritional assessment.

## 1 Introduction

Patients with head and neck malignancies often present with varying degrees of nutritional risk even before surgery due to tumor-related catabolism, impaired oral intake, and intense perioperative stress responses (1–4). Surgical trauma can further exacerbate the catabolic state, suppress visceral protein synthesis, and thereby negatively affect postoperative recovery and prognosis. Serum albumin has traditionally been used as a nutritional and prognostic marker, but its long half-life and susceptibility to inflammation and fluid status limit its sensitivity for short-term perioperative assessment.

In contrast, prealbumin (PA) and retinol-binding protein (RBP), which have shorter half-lives, may be more suitable for reflecting short-term changes in visceral protein status during the perioperative period, although both markers should be interpreted together with inflammatory indicators such as CRP (5).

Previous studies and clinical guidelines have emphasized the importance of nutritional screening, individualized nutritional support, and early intervention in patients with cancer and head and neck malignancies. However, evidence remains limited regarding the dynamic perioperative changes in multiple visceral protein markers and their relationship with different nutritional support strategies. Therefore, this study compared conventional and intensive nutritional support strategies and simultaneously analyzed the dynamic changes in ALB, PA, and RBP in combination with inflammatory markers, with the aim of providing more clinically relevant evidence for perioperative nutritional management (6–9).

## 2 Materials and methods

### 2.1 General information

A total of 200 patients with head and neck malignant tumors who underwent surgical treatment in the Department of Oral and Maxillofacial Surgery of our hospital from January 2023 to December 2024 were enrolled in this study. They were divided into a control group and an intervention group, with 100 cases in each group, according to different nutritional support pathways. This study was a single-center retrospective cohort study. Grouping was based on the actual nutritional management pathways received by patients during hospitalization rather than random allocation.

Inclusion criteria were elective surgery under general anesthesia and no administration of albumin preparations or systemic parenteral nutrition within 7 days before surgery.

**Figure 1:**
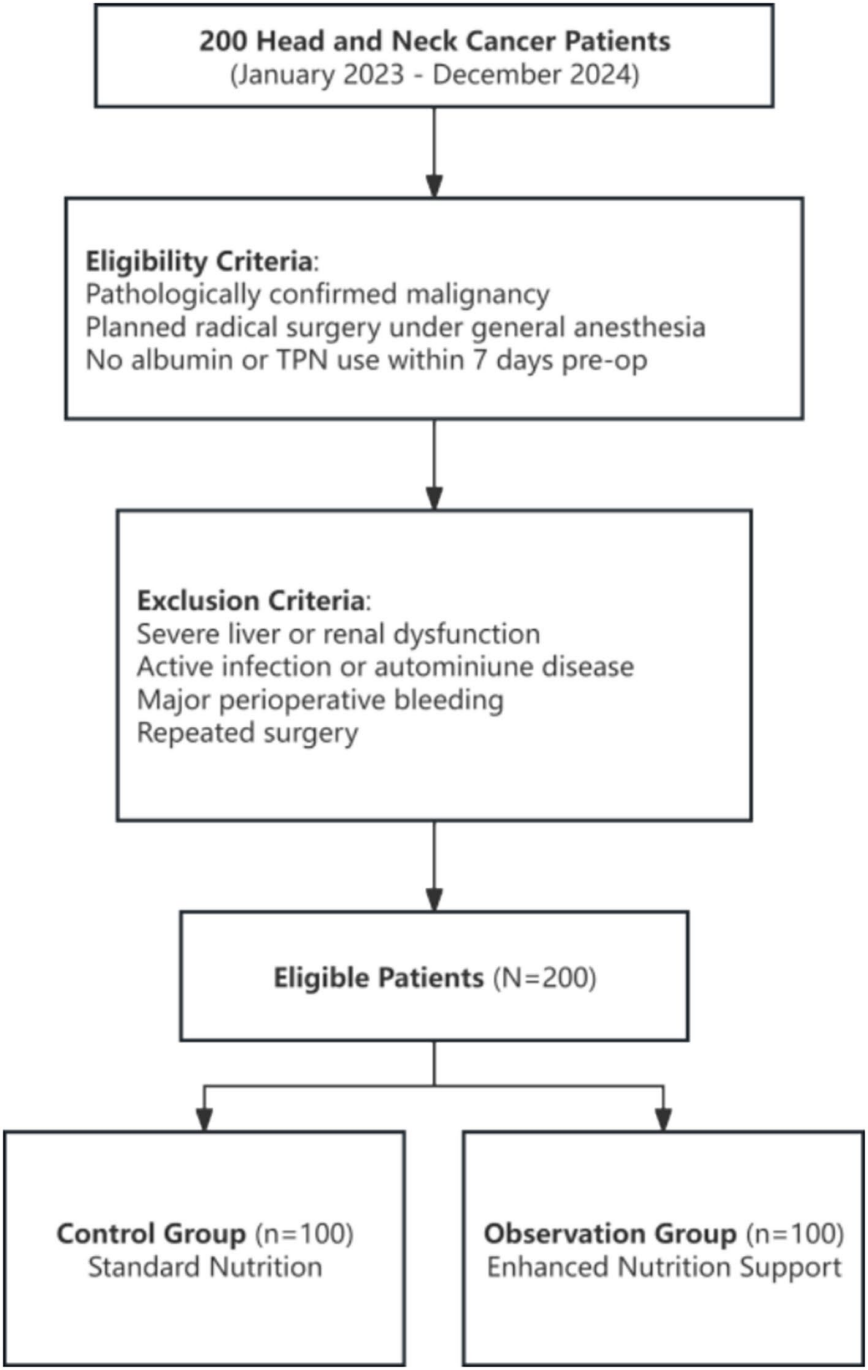
Flowchart of patient enrollment and group allocation.

Exclusion criteria were severe hepatic or renal dysfunction, active infection or immune-related disease, massive perioperative hemorrhage, or reoperation (Figure 1).

Baseline characteristics were comparable between groups (*p* > 0.05) (Table 1). Nutritional risk was assessed preoperatively using the Nutritional Risk Screening 2002 (NRS2002) tool. No significant difference in baseline nutritional risk was observed between the two groups.

**Table 1:** Baseline characteristics of patients.

Variable	Control (*n =* 100)	Intervention (*n =* 100)	*t*/*χ*^2^	*P*
Age (years, mean±SD)	56.47 ± 10.12	55.92 ± 9.76	*t =* 0.356	0.722
Sex (M/F), n	71/29	74/26	*χ*^2^ = 0.207	0.649
BMI (kg/m^2^, mean±SD)	22.08 ± 3.09	22.24 ± 3.14	*t =* −0.382	0.703
NRS2002 (score, mean±SD)	3.21 ± 0.84	3.15 ± 0.81	*t =* 0.487	0.627
Tumor site, *n* (%)			*χ*^2^ = 0.894	0.925
Oral cavity	33 (33.0)	35 (35.0)		
Oropharynx	19 (19.0)	17 (17.0)		
Larynx	24 (24.0)	22 (22.0)		
Hypopharynx	16 (16.0)	15 (15.0)		
Other	8 (8.0)	11 (11.0)		
Tumor stage (I–IV), *n* (%)			*χ*^2^ = 0.736	0.947
I	14 (14.0)	12 (12.0)		
II	27 (27.0)	29 (29.0)		
III	34 (34.0)	33 (33.0)		
IV	25 (25.0)	26 (26.0)		
Comorbidities, *n* (%)				
DM	18 (18.0)	16 (16.0)	*χ*^2^ = 0.150	0.698
HTN	31 (31.0)	34 (34.0)	*χ*^2^ = 0.196	0.658
Tracheostomy, *n* (%)	27 (27.0)	25 (25.0)	*χ*^2^ = 0.102	0.750
Operation time (min, mean ± SD)	318.42 ± 72.35	323.18 ± 74.62	*t =* −0.472	0.637
Blood loss (mL, mean ± SD)	435.60 ± 185.72	418.25 ± 178.43	*t =* 0.696	0.487
Reconstruction, *n* (%)			*χ*^2^ = 0.312	0.856
Free flap	41 (41.0)	43 (43.0)		
Pedicled flap	22 (22.0)	20 (20.0)		
None	37 (37.0)	37 (37.0)		

### 2.2 Methods

#### 2.2.1 Control group

Patients in the control group received routine perioperative care and conventional nutritional support. No structured preoperative nutritional assessment or planned nutritional intervention was implemented; instead, patients received general dietary advice. After surgery, oral intake was resumed gradually as gastrointestinal function recovered. When oral intake was not feasible or was insufficient, patients received conventional nutritional support.

Enteral nutrition was provided using a whole-protein enteral nutrition emulsion (generic name: enteral nutrition emulsion [TPF]; manufacturer: Nutricia Pharmaceutical (Wuxi) Co., Ltd.; National Drug Approval No. H20030011; specification: 500 mL, 1.5 kcal/mL), administered orally or via a nasogastric tube. Given that parenteral nutrition was necessary, the significant parenteral nutrition mainly included compound amino acid injection (18AA) (manufacturer: Shijiazhuang No. 4 Pharmaceutical Co., Ltd.; National Drug Approval No. H20013240; specification: 500 mL, 25 g), fat emulsion injection (C14-24) (manufacturer: Sichuan Kelun Pharmaceutical Co., Ltd.; National Drug Approval No. H20067140; specification: 250 mL, 75 g), and the relevant glucose injection, administered via peripheral or central venous routes. Furthermore, clinicians empirically adjusted the dosage and duration of nutritional support based on the patient’s general condition; no predefined energy or protein targets were specified, and nutritional adequacy was not monitored dynamically.

#### 2.2.2 Intervention group

Patients in the intervention group received intensive nutritional intervention in addition to routine perioperative management. Nutritional risk screening and comprehensive assessment were performed immediately after enrollment, and an individualized nutritional plan was developed based on body weight, BMI, and NRS 2002 score. Target energy intake was set at 25–30 kcal/(kg·day), and target protein intake was 1.2–1.5 g/(kg·day). Oral nutritional supplementation was provided when feasible to improve preoperative nutritional reserves.

In hemodynamically stable patients, enteral nutrition was initiated within 24 h after surgery using continuous infusion, starting at a low rate and advancing according to gastrointestinal tolerance, with the goal of achieving target energy intake within 3–5 postoperative days. Supplemental parenteral nutrition was added when enteral nutrition provided <60% of the target energy intake, and combined parenteral nutrition was used when necessary to meet energy and protein targets. Nutritional support was adjusted dynamically based on serial serum nutritional indices and inflammatory markers.

For the intervention group, daily energy and protein intake were retrospectively calculated based on a combination of medical records, nursing documentation, and dietary logs. Oral intake was recorded by nursing staff at each meal, including volume and type of food consumed, and converted into kilocalories and grams of protein using standardized nutrient composition tables. Enteral nutrition administered via tube feeding was documented in the electronic medical record, including infusion rate, total volume, and protein content. When parenteral nutrition was provided, the prescribed composition and infusion rates were recorded. For each patient, total daily energy and protein intake was calculated as the sum of oral, enteral, and parenteral sources. Missing or incomplete entries were verified and supplemented by reviewing physician and nursing notes where available. This approach ensured that daily caloric and protein intake was quantified as accurately as possible despite the retrospective study design.

### 2.3 Observational indicators

#### 2.3.1 Nutrition-related serological indicators

Fasting venous blood samples were collected in the morning, 1 day before surgery, and on postoperative day 7. Serum albumin (ALB), prealbumin (PA), and retinol-binding protein (RBP) were measured to evaluate perioperative changes in visceral protein status.

#### 2.3.2 Inflammation- and stress-related indicators

At the same time points, serum C-reactive protein (CRP), peripheral white blood cell count, and neutrophil percentage were measured. The neutrophil-to-lymphocyte ratio (NLR) was calculated as an index of systemic inflammatory response.

#### 2.3.3 Perioperative recovery and prognosis-related indicators

Postoperative time to first oral intake, length of hospital stay, and perioperative complications (including surgical site infection and pulmonary infection) were recorded to evaluate early postoperative recovery. Surgical site infection and pulmonary infection were diagnosed according to institutional criteria documented in the medical records.

Gastrointestinal tolerance was assessed based on abdominal distension, nausea, vomiting, diarrhea, abdominal pain, aspiration risk, gastric residual volume when applicable, and whether enteral feeding could be continued or advanced without interruption. Refeeding syndrome was evaluated according to newly developed hypophosphatemia, hypokalemia, hypomagnesemia, fluid overload, and related clinical symptoms after initiation of nutritional support. Feeding tube–related complications included tube obstruction, displacement, aspiration, nasal or pharyngeal discomfort, and feeding interruption caused by tube-related problems.

### 2.4 Statistical analysis

All statistical analyses were performed using SPSS (version 26.0). Continuous variables were assessed for normality and were summarized as mean ± standard deviation (SD) when normally distributed. Between-group comparisons were conducted using the independent-samples *t*-test, and within-group (pre- vs. postoperative) comparisons were performed using the paired *t*-test. For non-normally distributed continuous variables, the Mann–Whitney U test (between groups) or Wilcoxon signed-rank test (within groups) was used, as appropriate. Categorical variables were summarized as numbers and percentages [*n* (%)], and were compared using the chi-square test or Fisher’s exact test, as appropriate. A two-way analysis of variance (ANOVA) with a group × time factor was used to evaluate interaction effects. All tests were two-sided, and *p* < 0.05 was considered statistically significant. Cases with incomplete dietary intake records were reviewed using physician and nursing documentation whenever available. Patients with missing key nutritional intake data that could not be verified were excluded from the corresponding nutritional target achievement analyses.

To reduce potential confounding inherent to the retrospective observational design, multivariable linear regression analyses were performed. Postoperative ALB, PA, and RBP levels were used as dependent variables to evaluate the independent association between intensive nutritional intervention and postoperative nutritional biomarker recovery. Variables considered clinically relevant, including age, tumor stage, reconstruction type, operation time, and nutritional intervention strategy, were included as covariates.

Additional multivariable linear regression analysis was performed to identify factors associated with time to first oral intake. Nutritional intervention strategy, reconstruction type, age, tumor stage, and operation time were included as covariates. Regression coefficients (*β*) with 95% confidence intervals (CIs) were reported. All tests were two-sided, and *p* < 0.05 was considered statistically significant.

## 3 Results

### 3.1 Comparison of serum ALB levels before surgery and 1week after surgery between the two groups

There was no significant difference in serum albumin (ALB) levels between the two groups before surgery (*p* > 0.05). One week after surgery, the serum ALB level in the intervention group was significantly higher than that in the control group (*p* < 0.05), indicating a statistically significant difference (see Table 2).

**Table 2:** Comparison of serum albumin (ALB) levels before surgery and 1 week after surgery between the two groups (g/L, x̄ ± s).

Indicator	Control group (*n =* 100)	intervention group (*n =* 100)	*t*/*χ*^2^ value	*p*-value
Preoperative	41.18 ± 3.92	41.05 ± 3.87	0.229	0.819
One week after surgery	34.76 ± 3.58	36.82 ± 3.61	−4.048	< 0.001
ALB recovery rate (%)	84.40 ± 7.25	89.60 ± 6.83	−5.232	< 0.001
ALB < 35 g/L (%)	58.0	41.0	*χ*^2^ = 5.807	0.016
ALB within normal range (%)	42.0	59.0	*χ*^2^ = 5.807	0.016

### 3.2 Comparison of serum PA levels before surgery and 1 week after surgery between the two groups

There was no significant difference in serum prealbumin (PA) levels between the two groups before surgery (*p* > 0.05). One week after surgery, the serum PA level in the intervention group was significantly higher than that in the control group (*p* < 0.05), and the difference was statistically significant (see Table 3).

**Table 3:** Comparison of serum prealbumin (PA) levels before surgery and 1 week after surgery between the two groups (mg/L, x̄ ± s).

Indicator	Control group (*n =* 100)	intervention group (*n =* 100)	*t*/*χ*^2^ value	*P*-value
Preoperative	245.80 ± 41.95	247.60 ± 42.38	0.298	0.766
One week after surgery	174.12 ± 38.94	198.74 ± 41.26	−4.414	< 0.001
PA ≥ 200 mg/L (%)	38.0	54.0	*χ*^2^ = 5.120	0.024
PA < 150 mg/L (%)	35.0	22.0	*χ*^2^ = 4.067	0.044
PA recovery rate (%)	70.90 ± 10.82	80.30 ± 10.15	−6.230	< 0.001

### 3.3 Comparison of serum RBP levels before surgery and 1 week after surgery between the two groups

There was no significant difference in serum retinol-binding protein (RBP) levels between the two groups before surgery (*p* > 0.05). One week after surgery, the serum RBP level in the intervention group was significantly higher than that in the control group (*p* < 0.05), and the difference was statistically significant (Table 4).

**Table 4:** Comparison of serum retinol-binding protein (RBP) levels before surgery and 1 week after surgery between the two groups (mg/L, x̄ ± s).

Indicator	Control group (*n =* 100)	intervention group (*n =* 100)	*t*/*χ*^2^ value	*P*-value
Preoperative	43.38 ± 7.88	43.71 ± 7.75	−0.305	0.761
One week after surgery	29.41 ± 7.33	33.27 ± 7.85	−3.598	< 0.001
RBP ≥ 30 mg/L (%)	48.0	64.0	*χ*^2^ = 5.120	0.024
RBP < 25 mg/L (%)	30.0	19.0	*χ*^2^ = 3.419	0.064
RBP recovery rate (%)	67.80 ± 9.60	76.10 ± 9.25	−6.214	< 0.001

### 3.4 Comparison of inflammatory responses and postoperative recovery indicators between the two groups

**Figure 2:**
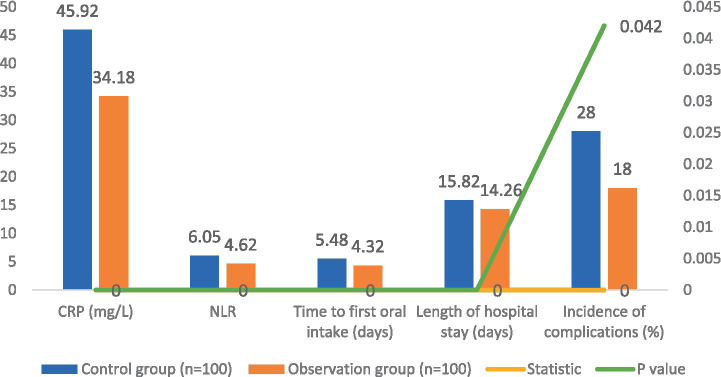
Comparison of inflammatory responses and postoperative recovery indicators between the two groups.

One week after surgery, levels of CRP and NLR in the intervention group were lower than those in the control group. In addition, the intervention group showed a shorter time to first oral intake, a shorter hospital stay, and a lower incidence of postoperative complications. All differences were statistically significant (*p* < 0.05) (see Table 5 and Figure 2). No cases of refeeding syndrome or feeding tube–related complications were observed in either the control or intervention group during the study period. Therefore, intensive nutritional intervention did not appear to increase the risk of these complications in our cohort.

**Table 5:** Comparison of inflammatory responses and postoperative recovery indicators between the two groups.

Indicator	Control group (*n =* 100)	intervention group (*n =* 100)	Statistic	*P*-value
CRP (mg/L)	45.92 ± 14.85	34.18 ± 13.42	*t =* 5.660	< 0.001
NLR	6.05 ± 1.88	4.62 ± 1.72	*t =* 5.395	< 0.001
Time to first oral intake (days)	5.48 ± 1.39	4.32 ± 1.28	*t =* 6.061	< 0.001
Length of hospital stay (days)	15.82 ± 3.54	14.26 ± 3.18	*t =* 3.228	< 0.001
Incidence of complications (%)	28.0	18.0	*χ*^2^ = 2.857	0.042

To further account for the influence of systemic inflammation on visceral protein levels, we calculated the ratios of ALB, PA, and RBP to C-reactive protein (CRP) for each patient. One week after surgery, the intervention group showed significantly higher PA/CRP and RBP/CRP ratios compared with the control group (*p* < 0.05), suggesting improved anabolic recovery independent of inflammatory response. The ALB/CRP ratio showed a similar trend, although differences did not reach statistical significance.

### 3.5 Comparison of postoperative nutritional marker-to-CRP ratios between the two groups

Ratios of PA/CRP and RBP/CRP were significantly higher in the intervention group than in the control group (*p* < 0.001), while ALB/CRP showed a nonsignificant increase (*p* = 0.083), suggesting improved nutritional recovery independent of inflammation (see Table 6).

**Table 6:** Comparison of postoperative nutritional marker-to-CRP ratios at postoperative day 7.

Indicator	Control group (*n =* 100)	Intervention group (*n =* 100)	*t*-value	*P*-value
ALB/CRP	0.79 ± 0.31	0.87 ± 0.34	−1.74	0.083
PA/CRP	4.12 ± 1.86	5.82 ± 2.14	−5.99	<0.001
RBP/CRP	0.69 ± 0.29	0.97 ± 0.35	−6.17	<0.001

### 3.6 Comparison of postoperative nutritional target achievement between the two groups

**Figure 3:**
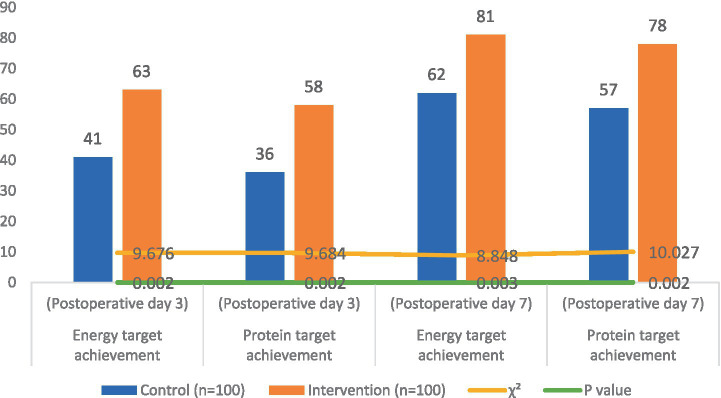
Comparison of postoperative nutritional target achievement between the two groups [*n* (%)].

On postoperative days 3 and 7, both the energy and protein target achievement rates in the intervention group were higher than those in the control group, and the differences were statistically significant (*p* < 0.05) (see Table 7 and Figure 3). These results suggest that intensive nutritional intervention is more effective in helping patients achieve the predefined nutritional targets during the early postoperative period.

**Table 7:** Comparison of postoperative nutritional target achievement between the two groups [*n* (%)].

Variable	Control (*n =* 100)	Intervention (*n =* 100)	*χ* ^2^	*P-*value
Energy target achievement (Postoperative day 3)	41 (41.0)	63 (63.0)	9.676	0.002
Protein target achievement (Postoperative day 3)	36 (36.0)	58 (58.0)	9.684	0.002
Energy target achievement (Postoperative day 7)	62 (62.0)	81 (81.0)	8.848	0.003
Protein target achievement (Postoperative day 7)	57 (57.0)	78 (78.0)	10.027	0.002

### 3.7 Multivariable regression analysis

To further evaluate whether intensive nutritional intervention was independently associated with postoperative nutritional recovery, multivariable linear regression analyses were performed using postoperative ALB, PA, and RBP levels as dependent variables. Age, tumor stage, reconstruction type, operation time, and nutritional intervention strategy were entered into the models.

After adjustment for potential confounders, intensive nutritional intervention remained independently associated with higher postoperative ALB (*β* = 1.98, *p* < 0.001), PA (*β* = 22.45, *p* < 0.001), and RBP levels (*β* = 3.41, *p* < 0.001). Advanced tumor stage was associated with poorer postoperative nutritional biomarker recovery, whereas longer operation time showed a modest negative association with postoperative nutritional status. Detailed results are presented in Table 8.

**Table 8:** Multivariable linear regression analysis of factors associated with postoperative nutritional biomarkers.

Variable	Postoperative ALB *β* (95% CI)	*P*-value	Postoperative PA *β* (95% CI)	*P-*value	Postoperative RBP *β* (95% CI)	*P-*value
Intensive nutritional intervention	1.98 (0.95–3.01)	<0.001	22.45 (12.37–32.53)	<0.001	3.41 (1.62–5.20)	<0.001
Age (years)	−0.03 (−0.06–0.00)	0.041	−0.42 (−0.79 to −0.05)	0.027	-0.05 (−0.11–0.01)	0.082
Tumor stage (III–IV vs. I–II)	−1.24 (−2.33 to −0.15)	0.026	−13.82 (−24.91 to −2.73)	0.015	−2.07 (−3.94 to −0.20)	0.030
Free flap reconstruction	0.88 (−0.12–1.88)	0.084	8.36 (−1.54–18.26)	0.097	1.12 (−0.55–2.79)	0.187
Operation time (min)	−0.01 (−0.02–0.00)	0.048	−0.09 (−0.18–0.00)	0.044	−0.02 (−0.03–0.00)	0.051

Linear regression showed that both intensive nutritional intervention and reconstruction type independently affected time to first oral intake, with free flap associated with longer delay (Table 9).

**Table 9:** Multivariable linear regression of factors associated with time to first oral intake.

Variable	*β* (95% CI)	*P*-value
Intervention group (vs. control)	−1.12 (−1.64, −0.60)	<0.001
Free flap reconstruction (vs. pedicled flap)	0.74 (0.12, 1.36)	0.020
Age (years)	0.02 (−0.01, 0.05)	0.210
Tumor stage III–IV (vs. I–II)	0.58 (0.05, 1.11)	0.032

## 4 Discussion

### 4.1 Characteristics of perioperative nutritional status in patients with head and neck malignant tumors

In this retrospective cohort study, intensive perioperative nutritional intervention was associated with improved early postoperative visceral protein recovery and reduced inflammatory markers after head and neck cancer surgery. Although baseline nutritional status was comparable between the two groups, patients generally experienced a decline in nutrition-related indicators within 1 week after surgery. These findings indicate that the early postoperative period represents a high-risk stage for rapid deterioration of nutritional status.

This deterioration may be attributed to surgical trauma, systemic inflammatory activation, and neuroendocrine stress responses. These factors can shift metabolism from an anabolic state toward a catabolic state, thereby increasing protein breakdown and suppressing visceral protein synthesis (10, 11). In addition, head and neck surgery often leads to swallowing pain, restricted oral intake, airway management requirements, and psychological stress, making it difficult for patients to meet energy and protein requirements through spontaneous oral intake in the short term.

The combined effects of these factors may explain why patients with head and neck malignancies are particularly vulnerable to perioperative malnutrition. From a dynamic perspective, the present findings support the need for structured perioperative nutritional management rather than reliance on routine care or spontaneous postoperative recovery alone.

### 4.2 Clinical value of serum ALB, PA, and RBP in perioperative nutritional assessment

Serum albumin has long been used as a marker of nutritional status and clinical prognosis because of its relative stability and its ability to reflect overall disease severity. However, albumin is also influenced by non-nutritional factors during the perioperative period, including inflammation, fluid shifts, hemodilution, and changes in vascular permeability (12). Therefore, albumin may respond slowly to short-term nutritional intervention and has limitations when used alone for early postoperative nutritional assessment.

Compared with albumin, prealbumin and retinol-binding protein have shorter half-lives and may be more sensitive to short-term changes in energy and protein supply. However, these markers are also influenced by infection, inflammation, liver function, and renal function, and should therefore be interpreted together with CRP or other inflammatory indicators.

However, PA and RBP are also negative acute-phase proteins, and their levels may be suppressed by systemic inflammation. Therefore, these indicators should not be interpreted in isolation, and nutritional marker-to-CRP ratios may provide complementary information (13).

### 4.3 Effects of intensive nutritional intervention on postoperative nutritional recovery and prognosis

In this study, intensive nutritional intervention was associated with improved early postoperative visceral protein recovery, lower inflammatory markers, and better short-term clinical recovery. The main features of the intervention included early nutritional risk assessment, individualized energy and protein targets, early initiation of enteral nutrition when clinically feasible, and dynamic adjustment according to gastrointestinal tolerance and laboratory indicators (10, 14–16).

Adequate energy intake may reduce endogenous protein catabolism during the postoperative stress response, while sufficient protein and amino acid supply may support visceral protein synthesis, wound healing, tissue repair, and immune recovery. Early enteral nutrition may also help maintain intestinal mucosal barrier function, reduce inflammatory amplification, and improve nutritional delivery during the early postoperative period (11, 17, 18). The association between intensive nutritional intervention and shorter time to first oral intake should also be interpreted in the context of surgical complexity and perioperative recovery pathways, particularly in patients undergoing free flap reconstruction (19–22).

### 4.4 Limitations

This study has several limitations. First, this was a single-center retrospective cohort study. Although the sample size was expanded, a certain degree of selection bias remains, and the non-randomized group allocation may limit causal inference. Although preoperative nutritional risk was assessed using NRS2002, formal malnutrition diagnosis according to the GLIM criteria was not performed in all patients because of the retrospective nature of the study and incomplete availability of body composition and muscle mass data (23).

Second, although several baseline characteristics were included and compared, potential confounding factors such as tumor site, tumor stage, surgical approach, swallowing function, and postoperative rehabilitation may still influence postoperative nutritional recovery and clinical outcomes. In addition, detailed pathological variables, including tumor size, exact subsite, and histological grading, were not consistently available for all patients in the retrospective records and therefore could not be incorporated into the multivariable models.

Different reconstruction procedures, particularly free flap versus pedicled flap reconstruction, may independently influence postoperative swallowing recovery and the timing of first oral intake. In response to this concern, reconstruction type was included in the additional multivariable regression analysis for time to first oral intake. However, residual confounding related to surgical complexity, postoperative swallowing function, rehabilitation strategy, and surgeon-specific practice patterns could not be fully excluded.

Third, the retrospective assessment of daily energy and protein intake may be affected by incomplete dietary records, particularly for spontaneous oral intake. Although dietary logs, nursing documentation, enteral nutrition records, parenteral nutrition prescriptions, and physician notes were reviewed together whenever possible, the accuracy of retrospective intake estimation remains lower than that of prospective dietary assessment.

Fourth, ALB, PA, and RBP are all negative acute-phase proteins, and their levels are susceptible to inflammatory status. To better isolate the nutritional effect from the postoperative systemic inflammatory response, we included the ratios of ALB, PA, and RBP to CRP in the Results section. However, although CRP and NLR were incorporated into the analysis, it remains difficult to fully distinguish the relative contributions of nutritional improvement and inflammatory resolution.

Finally, the observation period was limited to 1 week after surgery, which precluded evaluation of long-term nutritional status, quality of life, treatment tolerance, and long-term prognosis. Therefore, future multicenter, prospective studies with standardized nutritional protocols, validated malnutrition assessment, and longer follow-up are warranted to validate these findings further.

## 5 Conclusion

Intensive perioperative nutritional intervention was associated with improved early postoperative recovery of serum albumin, prealbumin, and retinol-binding protein after surgery for head and neck malignancies, together with lower inflammatory markers and higher nutritional target achievement rates. These changes were accompanied by shorter time to first oral intake, reduced length of hospital stay, and a lower observed incidence of postoperative complications. PA and RBP appeared more sensitive than ALB for dynamic perioperative nutritional monitoring. Prospective studies with standardized nutritional protocols, surgical stratification, and longer follow-up are warranted to confirm these findings.

## Data Availability

The raw data supporting the conclusions of this article will be made available by the authors, without undue reservation.
